# How did healthcare professionals define patient engagement in quality management? A survey study

**DOI:** 10.1186/s12913-023-09098-z

**Published:** 2023-02-20

**Authors:** Ana Maria Saut, Linda Lee Ho, Simone Berger, Fernando Tobal Berssaneti

**Affiliations:** 1 Polytechnic School - Production Engineering Department, University of São Paulo (USP), Av. Prof. Luciano Gualberto, 1380, São Paulo, 05508-010 Brazil; 2 Engineering School - Production Engineering Coordination, Mackenzie Presbyterian University, São Paulo, Brazil

**Keywords:** Patient Safety, Quality of Health Care, Quality improvement, Patient Participation

## Abstract

**Background:**

Patient and family engagement (PFE) can positively impact the patient experience and care process outcomes. There is no unique type of PFE, and the process is usually defined by the quality management department or professionals responsible for this process in the hospital. The objective of this study is to define PFE in quality management based on the professional’s perspective.

**Method:**

A survey was carried out with 90 professionals from Brazilian hospitals. There were two questions aimed at understanding the concept. The first was a multiple-choice question to identify synonyms. The second was an open-ended question to develop the definition. A content analysis methodology was employed by applying techniques for thematic and inferential analysis.

**Results:**

Three words were classified as synonyms by more than 60% of respondents: involvement, participation, and centered care. The participants described patient participation at both the individual (related to the treatment) and organizational levels (related to quality improvement). The PFE in the treatment is related to the development, discussion and decision-making about the therapeutic plan, participation in each step of care, and knowledge of the institution's quality and safety processes. At the organizational level, engagement in quality improvement includes the involvement of the P/F in all processes of the institution, from strategic planning to the design or improvement processes, as well as active participation in institutional committees or commissions.

**Conclusion:**

The professionals defined engagement in two levels (individual and organizational) and the results suggest that their point of view can influence the practice in the hospitals. Professionals of hospitals that implemented mechanisms of consult defined PFE more in the individual level. On the other hand, professionals of hospitals that implemented mechanisms of involvement considered PFE more focus in the organizational level.

**Supplementary Information:**

The online version contains supplementary material available at 10.1186/s12913-023-09098-z.

## Introduction

The growing global importance of patient safety and improving the quality of health services has generated interest in the theme of patient and family engagement (PFE). According to Donald Berwick, we are facing the third era of quality healthcare that emphasizes the importance of the active voices of patients [1]. The patients can be considered "specialists by experience" [2]. Hospitals have established objectives that value the patient’s perspective [3]; however, the inconsistency and lack of consensus about the concept of 'patient engagement' represents a knowledge gap [4–10].

The conceptual gap has been discussed in the literature reviews on the subject for more than two decades. The published reviews were from recognized journals, which evidenced the same problem. In 1998, Cahill had already identified different concepts in the literature and observed the need to formulate a standardized definition [11]. Longtin et al. (2010) concluded in their review that there was no single definition and that several terms were used interchangeably [5]. Mockford et al*.* (2012) observed that the studies rarely provided an explicit definition and lacked consistency [6], a scenario that remained in later reviews [7–10].

According to Castro et al. (2016), the absence of theoretical and conceptual clarity has compromised the understanding and communication between researchers, health professionals and policy-makers, in addition to generating problems in measuring and comparing the results of studies conducted in different institutions [4].

These deficiencies have impeded the diffusion of theory in the practice of health services. Patient involvement is dictated by available opportunities and resources. Healthcare professionals must maximize the potential and opportunities for patient involvement, whereas patients have become essential for high-quality healthcare service provision [12].

Anderson et al. (2022) confirmed the positive impacts of patient engagement, patient/family advisor and clinician/staff capacity to undertake PFE and the impacts of PFE outputs on clinician/staff function and processes, patient experience, and patient care outcomes [13]. Involving staff is one of the most challenging aspects in the promotion of a favorable environment to engage patients. People are more likely to act if they believe [14].

The involvement of patients and the community can have an impact on the "planning and development" of services, the “development, information development and dissemination” and the “changing attitudes of service users and providers" [6]. The creation of collaborative partnerships between users and service providers can contribute to the coplanning and coimplementation of innovative service models [9].

As stated by Saut et al. [15], effective patient engagement involves changes in both process and culture. Patient engagement must be addressed in a more comprehensive and integrated way, considering all the essential elements related to processes and organizational culture. There is no unique form to do this. The engagement mechanisms are defined and designed by the quality management department or usually by the administrative area when this first one is not implemented. According to Rasheed et al*.*, institutions' internal actors need to be able to act as transforming leaders in change initiatives and quality improvement projects [16].

For Juran [17], considered as one of the gurus of quality, the word quality has multiple meanings, but two dominate the term: quality consists of the characteristics of the product or service, which meet customer needs and thus provide satisfaction with the product, and quality consists of being free from defects [18]. Quality management comprises three basic management processes, called ‘Juran's trilogy’: quality planning (the steps of establishing quality goals, identifying customers and their needs, developing product and processes, and establishing process controls); quality control (the actions of evaluating quality performance, comparing actual performance with quality goals, acting on the difference); and quality improvement (includes proving the need, establishing the infrastructure, identifying improvement projects, establishing project teams, providing teams with resources, training and motivation, diagnosing the causes and stimulating the implementation of solutions, in addition to establishing controls to maintain improvements). Quality improvement necessarily implies a consistent change in the quality level of the service provided.

In the healthcare sector, the Institute of Medicine (IoM) has identified six dimensions for healthcare quality: safe, effective, patient-centered, timely, efficient, and equitable [19]. To meet these six dimensions, Berwick, Nolan and Whittington proposed three objectives, known as the ‘triple aim’: to improve the care experience, improve the population's health and reduce the per capita costs of healthcare [20]. In 2015, Berwick, Feeley and Loehner expanded the concept by highlighting the importance of establishing a partnership with patients, health professionals, and communities [21].

Considering the lack of clarity in the definition and the importance of healthcare professionals in the planning and implementation of patient engagement mechanisms to improve service quality, the main objective of this research is to identify the definition of PFE based on the perspective of these professionals. To the best of our knowledge, no research on this topic has been carried out with professionals responsible for quality management in hospitals.

## Method

To achieve the objectives of this study, an exploratory study was carried out with a quantitative approach, applying the survey methodology. A web-based questionnaire survey was undertaken between August 2019 and May 2020. The population was hospitals located in Brazil. There were approximately 7,000 (426,000 beds) hospitals in Brazil. The invitation was sent to approximately 1,280 hospitals to participate in the study.

The invitation to answer the questionnaire was directed to the quality management area, and when there was no corporate area with this function, it was forwarded to a professional indicated by the institution with sufficient knowledge of the processes to answer it. There was only one respondent per hospital who could involve other professionals if deemed necessary.

The questionnaire comprised the following parts: a profile of organizations and respondents and two questions relating to the definition of patient engagement. The first was a multiple-choice question to identify synonyms. Eight choices were presented based on the literature review: activation [18, 22, 23], collaboration [5], coproduction [9, 24, 25], patient-centered care [19, 26, 27], empowerment [4], involvement [6, 28–30], partnership [5], and participation [4]. The second was an open-ended question to develop the definition: How would you define 'patient and family engagement in quality management'? To identify the hospital profile, the questionnaire had 24 mechanisms [3, 10, 31–35] (see Appendix 1) to support the identification of the level of engagement—consultation, involvement, partnership and shared leadership [31].

To analyze the answers to the open-ended question, a content analysis methodology was employed by applying techniques for thematic and inferential analysis [36]. It started with the preanalysis stage, which consisted of reading all the answers and constructing hypotheses, followed by the analysis of the word frequency and the relations between the words that support the identification of the themes, and finally, the development of the definition based on the themes. The content analysis was conducted using the software NVivo®12 (version 12.6.0.959 – plus edition) and Tropes (version 8.4.4).

The sampling was chosen by convenience, which is a nonrandom sample. Professionals from quality management (preferably, when it is possible) or other departments indicated by the institution with sufficient knowledge of the processes (for instance, Technical Director, Clinical Director, Nursing, Executive Board, Risk Management, Patient Safety or Ombudsman) were eligible to participate if they had a minimum of six months of experience in their current position. The respondents were invited by phone, email, or the contact form on the institutional websites. Additionally, National Accreditation Organization (ONA) supported the project and invited hospitals from its database. Reminders were sent thrice within an interval of 15–20 days to those who had not responded to the questionnaire.

Throughout the research, the word family is used to represent those people referred to as such by the patient, those he or she trusts and with whom he or she has a good relationship [37], including friends [38].

The Research Ethics Committee approved the project. The participants were informed of the objective of the study and signed the informed consent form. The questionnaires were answered online using SurveyMonkey® research software.

## Results

The final sample comprised 90 Brazilian professionals from hospitals. Most were professionals who had leadership or managerial positions (87.78% of respondents) and were female (74.44%). The average age was 41.9 years. Regarding education level, 98.89% graduated, with 92.22% also having a master or doctorate degree. The average time in the current position and working in the hospital are 5.72 years (SD 5.27) and 10.37 years (SD 8.56), respectively. Three quarters of respondents have at least 12 years of professional experience. The final sample of 90 hospitals represents a response rate of 7% (total invitations sent 1282).

Most of the institutions (hospitals) are general (72.22%), medium (41.11%) and large (37.78%). Approximately 79% perform educational functions. More than 80% reported that they had a quality department, and the respondents were from this area.

The classification of hospitals according to the level of engagement was based on the model proposed by Carman et al. [30] which resulted in approximately 75% consultation or less, 24% involvement and one percent partnership and shared leadership level.

An analysis was carried out to compare the profile between the hospitals in the sample and the population (the total number of hospitals located in Brazil) considering four variables, which are type of administrative control (for instance, public, private, beneficent or others), type of establishment (general, specialized, hospital day or others), location (distribution by region) and percentage of hospitals that perform an educational function. The result of an adherence test (also known as a goodness-of-fit test) showed that there was evidence that the probability distribution of the sample was the same as the population, regarding the type of administrative control (p-value = 0.814) and of type of establishment (p-value = 0.476). On the other hand, there was no evidence that the frequency distribution was the same for the other two characteristics evaluated – distribution by region (p-value < 0.001) and institution perform an educational function (p-value < 0.001). Approximately 76% of the hospitals in the sample reported that they perform an educational function, however the percentage in the population is around 9–10%.

### Synonyms

**Fig. 1:**
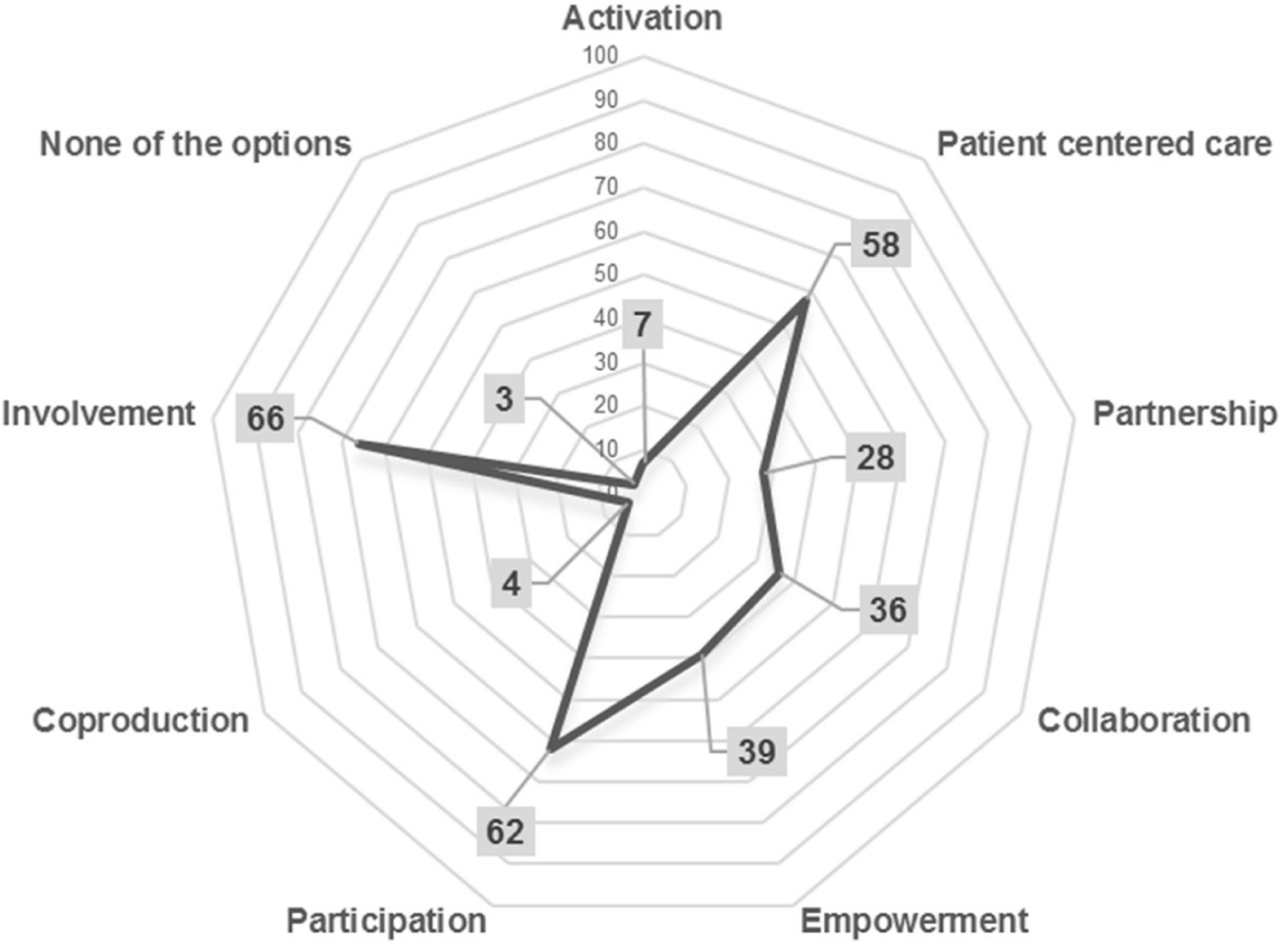
Chart of synonyms of the expression 'patient engagement' according to the number of respondents (*N* = 90). Source: The authors. Note: The numbers in the graph represent the total of respondents who classified the term as a synonym. The respondents could choose more than one option; for this reason, the sum of the total number of responses was greater than the sample size (90)

Figure 1 shows the frequency of words chosen as synonyms of patient engagement. More than half of the respondents selected at least one of the following three words as synonyms (average 3.33 words per respondent; SD 1.77): involvement (73.33%), participation (68.89%) and centered care (64.44%).

### Preanalysis of the definition of PFE

Concerning the definition, in the preanalysis, a high number of responses were observed that defined PFE as a process geared to the individual level of treatment (71 of 90 responses). The other responses addressed the definition of participation in quality management at the organizational level or at both the individual and organizational levels. It was also observed that some participants did not know how to define it: “I do not know how to define it”.

Related to the profile of respondents and institutions, most that reported not implementing a quality department defined engagement at the individual level. In terms of the level of engagement, 62% of the institutions that implement PFE at the consultation level (for instance, mechanisms such as survey of satisfaction or experience) defined at the individual level. A total of 71% with mechanisms at the involvement level (for example, hospitals that implement patients’ committees or involve them in improvement projects, advisory boards, training, and others) described PFE at the organizational level. It was not possible to identify differences between the answers according to the other characteristics of the respondents or institution profiles.

**Fig. 2:**
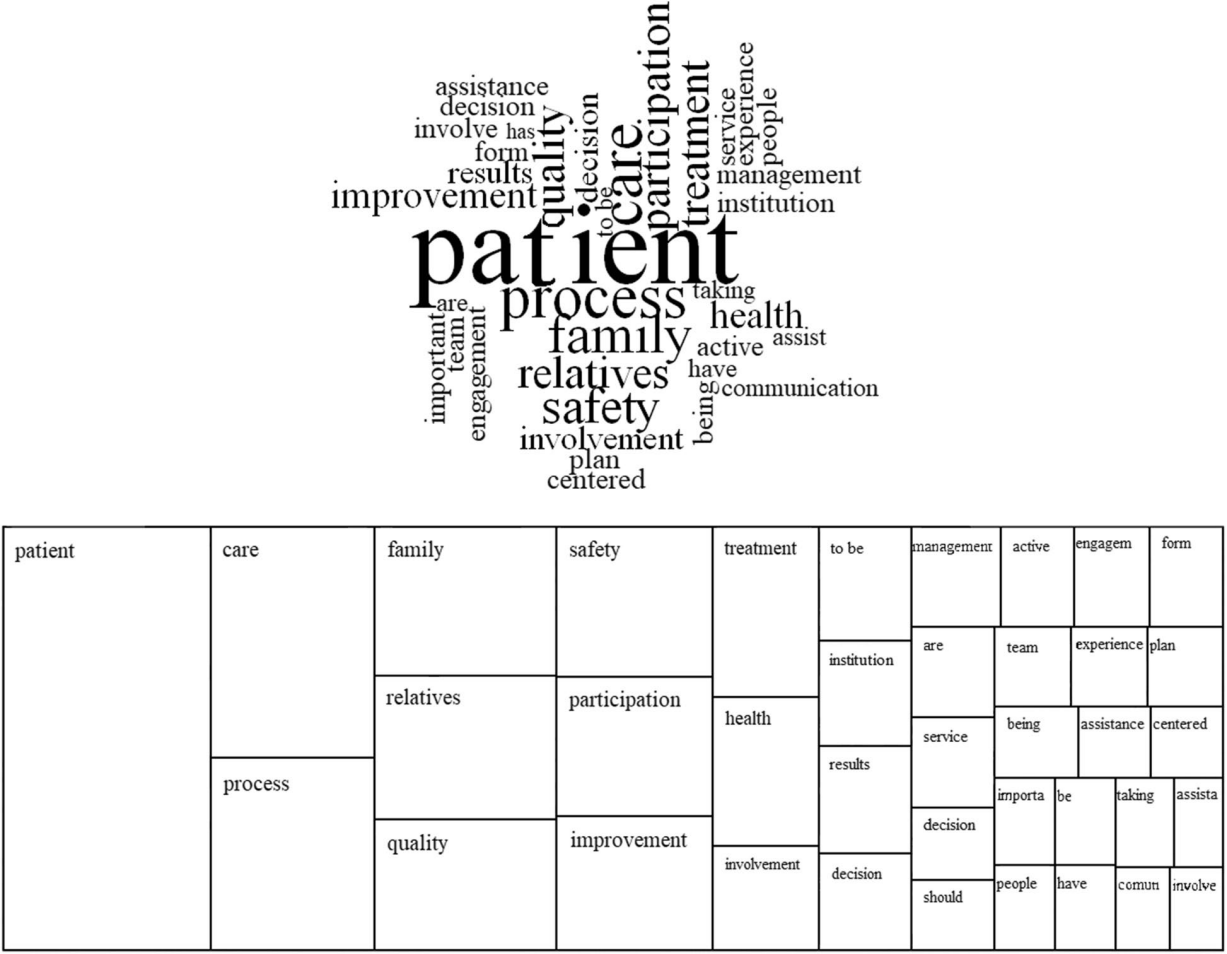
Word frequency cloud of patient engagement description. Source: NVivo® software. The size of the words in the cloud is proportional to the frequency of citations

In the second stage of exploring the answers, they were analyzed with the support of NVivo® and Tropes software. In the cloud of the most cited words, it was observed that the central word is 'patient', and two themes emerged: 'care', at the top, and 'processes', at the bottom (Fig. 2). From the map with the structure of the cloud, 'care' was related to safety and treatment, concerning the level of individual care. On the other hand, ‘processes’ were related to quality, improvement, participation, health, and institution, suggesting a reference to the organizational level.

The frequency of the words that most appeared in the answers were patients (118 citations), care (51 citations), process (42 citations), family (36 citations)/relatives (35 citations), quality (32 citations) and safety (31 citations). The words participation (30 citations) and involvement (15 citations) also appear with a high frequency, given that both were often cited as synonyms for engagement in the first question. This demonstrates that in the base repertoire, few new words were found, and in fact, the answers repeat some words of the question asked in the questionnaire.

In the next step, the most frequent relations between the words were analyzed to understand how they were used in the answers. In Tropes software, the relations analysis show which equivalent classes of the words (verbs, substantives, or others) are frequently connected (for instance, found in the same proposition) within the text analyzed. These relations underline the heart of the discourse: actors, objects, things and concepts presented in the text will appear before the readers in decreasing order of importance. In this study, some of the most cited words were mentioned to open the answer writing as patient ↔ family (27 citations); participation ↔ patient (19 citations); and participation ↔ family (12 citations). The others seem to define engagement based on the respondent’s perspective: patient ↔ care (18 citations); safety ↔ patient (16 citations); patient ↔ process (11 citations); active ↔ participation (8 citations); and centered ↔ care (6 citations).

To identify the themes and concepts that emerged from the answers, the excerpts that presented the relationships between the words were analyzed (see Table 1). Four categories were identified: what is, where, why and how to engage patients in service quality management. In the first category ‘What is’, engage was defined as participation. Related to ‘where’ or ‘on what’ engagement takes place, it was observed two concepts, one focused in the process of care (routine) and another in continuous quality improvement. The reasons (category ‘why’) for engaging were to guarantee patient safety and to promote patient centered care. Finally, the last category was ‘how’ to engage. P/F should participate in the institution’s processes at both individual and organizational level. It is necessary to establish formal processes to engage. To engage in quality improvement includes information gathering with P/F, involvement in care and improvement actions.

**Table 1:** Analysis of the relationships between the words used in the definition of engagement of patients and family members

Category	Theme/concept	Examples of excerpts	Comment
What is engage?	Participation and involvement are used as synonyms of engagement	"I believe it is the **participation** of the **patient** and the **family** in each stage of patient care and an understanding of the quality process implemented." **"Involvement of the patient and family** member in his or her treatment and interrelationship with the teams involved in his or her treatment seeking patient-centered care.”	Just as the answers to the closed question about the synonyms of engagement, the words ‘participation’ and ‘involvement’ were mentioned at the beginning of the definitions
Where can patients be engaged?	P/F participation in care	"We understand that the **patient** should be the center of **care** and participate in the development and conduction of his or her therapeutic plan.” "The participation of the **patient** and **family** member in all **care** tasks during his or her stay in the hospital.” "Involve the **patient** in the quality of his or her **care** and in his or her safety; actively participate in the process of their **care**.” "I believe it is the **participation** of the **patient** and the **family** in each stage of patient care and an understanding of the quality process implemented.”	Patient and family participating in all stages of care, to improve quality and safety
	P/F participation in continuous quality improvement	"[…]. With this, he (patient) becomes a fundamental agent in both the safety of his or her care and the **quality improvement**.” "The patient be engaged in all processes of the institution as part of continuous **improvement** of the **quality** provided to his or her treatment.” "Patient engagement in care is an important strategy for healthcare services **quality improvement** […] " It is one of the pillars of patient-centered care. It generates benefits in both levels individual and institutional, bringing **improvement** of **quality** and safety; improvement of care outcomes; increased satisfaction and loyalty of employees; improvement of the patient experience; improvement of time management; and communication between teams and patients/family members.”	P/F can contribute to continuously improving the quality of services
Why engages patient?	Patient safety as one of the expected results	"Patient-centered care; multidisciplinary team carry out guidance on **patient safety;** quality research; family involvement in patient care; and community participation in some institutional committees.” "In the last years, patient involvement has been recognized as an essential component in the health care process. WHO, through the World Alliance for Patient Safety**,** emphasizes the need to raise awareness among **patients** and their families about the importance of their engagement in initiatives to promote their own **safety** […]" "In my opinion, it is the education of the patient and family members concerning the **patient safety** goals, explaining, and demonstrating the reason for the processes involving **patient safety**, so that all people involved in care can contribute to safety and quality of care.”	The patient's participation in their care is important to ensure his or her safety
	Patient-centered care promotes and is strengthened by patient involvement in his or her treatment	"Engaging patients and family members in his or her treatment is very important to promote **patient-centered care**.” "It is one of the pillars of **patient-centered care** […] "	Both relationships always appear associated and could be rewritten as patient-centered care Engagement is important in seeking centered care and allows the patient participation
How	P/F as a participant in the institution's processes	"It is the **patient** being engaged in all **processes** of the institution as part of continuous improvement of the quality assistance during his or her treatment.” "The active participation of the **patient** and family not only in his or her own care, but in all **processes** of quality management of an institution, from the definition of strategic planning to the management of results and improvement projects.”	P/F participates in both care and quality management processes
	Active describes a characteristic of patient participation	**"Active participation** of **patients** and family members in the delivery of value, based on the experience and clinical outcome.” "An **active participation** through opinion, gesture or manifestation when making the decision about the institution, taking into account the care provided, or simply commenting at a time opinion regarding the service received when it is possible.”	Both relationships appear associated in most of the stretches, so it can be considered as active participation of the patient
	P/F engagement in processes to improve quality requires an organization (process definition)	"Involvement of the patient and family member in decision-making regarding their therapy in order to seek adherence, support or more appropriate strategies throughout the **process** in which the **patient,** caregiver or family member needs care.” "Have **processes** that facilitate the inclusion of **patients** and families in:(a) shared decision in treatment; (b) opportunities for improvement (ombudsman or customer service); (c) redesign of processes through improvement projects; and (d) advisory board.”	The word process appears in two contexts: 1. Process aimed at engaging patients 2. Engagement of patient in the care processes
	The expression quality management includes information gathering with P/F, involvement in care and improvement actions	"Patient and family engagement in **quality management** is the most powerful tool for achieving goals.” "Currently, our ombudsman focuses on **quality management;** in this way collecting data and transforming it into management information to improve the service provided. We are connected to a humanization working group and receive all users to collect manifestations (suggestions, compliments, complaints, and requests). The ombudsman is the voice of the user within the management.”	The expression 'quality management' has a comprehensive concept, in that it encompasses the processes of communication with the patient, their involvement in treatment and management actions, at the organizational level to improve quality and safety
	Quality and safety in care, at the individual and organizational level	"Involve the **patient** in the **quality** and safety of his or her care; actively participate in the process of his or her care.” "**Patient** engagement in care is an important strategy to improve the **quality** of healthcare services. Patients are a source of information for quality of care, corroborate the visibility of inequities and facilitate the design of strategies based and focused on results […]" "In general, **family** members are participatory, concerned with well-being of the patient and are cooperative with institutional **quality**.”	Words appear associated both to refer to the quality of care at the individual level (with emphasis on communication as a source of information), and to cooperate to improve quality and patient safety at the organizational level

Source: the authors. *P/F* patient and family, *WHO* World Health Organization

#### Definition of PFE at the individual level

As mentioned, some participants focused on the individual level to define PFE, which means involving them in their care and in the decision-making process about their therapeutic plan.

"Family and/or patient participation in the care given."

"Involvement of the patient and family in activities related to quality and safety of their care."

“To bring the patient to decisions related to their care interacting with the healthcare team.”

It was observed that at this level, professionals highlighted patient-centered care and the communication process between patients and professionals.

"Engaging patients and family members in his or her treatment is very important to promote patient-centered care."

"It is one of the pillars of patient-centered care [...]."

"[...] The proposal of patient-centered care allows the participation of the family/patient in the decision-making process [...]."

“Patient and family involvement in their treatment and interrelationship with the teams involved in their treatment in seeking patient-centered care.”

“Today, the entire process standardization service, as well as patient safety centers and customer relationship service, among others, are geared toward ensuring the quality of healthcare. All demands coming from the patient and their families are promptly heard and, as far as possible, their engagement is promoted.”

The main objective for involving patients seems to be the quality and safety process.

“In addition to healthcare professionals, family members and patients understand the safety steps.”

“The participation of the patient and family in care and safety, when they are part of decision-making and are also responsible for the process.”

#### Definition of PFE at the organizational level

At the organizational level, the participants mentioned the involvement of patients contributing to hospital outcomes and objectives as a whole.

“Care management integrated with the institution's objectives.”

“Patient and family engagement presupposes the participation of these people in the management of the hospital as a whole and not only in quality management.”

“Encouraging an environment in which the patient and his or her family perceive their responsibility and impact on the Institution's processes and results.”

The respondents mention the use of mechanisms for patient involvement. At the organizational level, engagement in quality improvement includes the involvement of the P/F in all processes of the institution, from strategic planning to the design of new processes or improvement of existing processes, as well as active participation in institutional committees or commissions.

“The participation of the patient and the family in the evaluation of the quality of the service provided, including suggestions, complaints, and praise, during or at the end of their treatment period.”

“Promotion of disclosure, involvement in the analysis of adverse events.”

“To have processes that facilitate the inclusion of patients and families in: a) shared decision-making in treatment, b) opportunities for improvement (Ombudsman or Customer Service), c) redesign of processes through improvement projects, and d) advisory board.”

Engagement also appears to be a strategy to improve the patient experience and the delivery of value for patients.

“The point of view of the patient and his or her family must be taken into account so that we can provide a less stressful experience for them and minimize the pain of a difficult time.”

“Active participation of patients and families in the delivery of value, based on experience and clinical outcome.”

As the result expected, at the organizational level, involvement can promote the continuous improvement of care processes.

“It is the involvement/interaction/relationship of clients in/with the continuous improvement of health services processes.”

“Currently, this is the major project that institutions should invest in it, as the involvement of the patient and their families makes an extremely positive contribution to the vision of continuous improvement that quality seeks to work on day-to-day in health units. Understanding the specificities of patients and families, giving them a voice, understanding the user's view of the processes brings different perspectives and focuses on seeking improvements of all processes.”

Table 2 summarizes the definition of patient and family engagement in quality management, considering both the individual and organizational levels. The results per level of engagement were compared considering the four categories presented in Table 1 (what is, where, why and how to engage patients in service quality management). The results demonstrate that the two levels encompass different processes, with different mechanisms, but have complementary objectives to guarantee the quality of services and patient safety.

**Table 2:** Definition of patient engagement at the individual and organizational levels

Where' or ‘on what’ engagement takes place	Individual level	Organizational level
What is engagement	The involvement of P/F in their care and in the decision-making process about their therapeutic plan	The involvement of P/F in contributing to hospital outcomes and objectives as a whole
How engagement is performed	In the communication process between patients and professionals	Through mechanisms involving P/F in all processes of the institution, from strategic planning to the design of new processes or improvement of existing processes, as well as active participation in institutional committees or commissions
Why engage	Involving patients in their care seems to improve the quality and safety process	To improve the patient experience, delivery of value for patients, and promote the continuous improvement of care processes

Source: the authors. *P/F* patient and family

## Discussion

The objective of this study was to define PFE in quality management based on the professional’s perspective. They defined two different perspectives. On the one hand, it was observed a definition considering the engagement in the individual level, which means patients engaged in their care and in the decision-making process about their therapeutic plan. On the other hand, PFE was also defined as the involvement of P/F in contributing to hospital outcomes and objectives as a whole.

First, the analysis of concepts and themes that emerged from the definitions was unfolded into four parts: what is, how, where and why to engage patients in service quality improvement. The results suggest that there is a relationship between the respondents' point of view about what engagement means and the level of engagement performed by institutions according to the mechanisms they have implemented.

'What is engagement’ corroborates the description proposed by Herrin et al. [32] in which PFE covers several related concepts, all based on the idea of involving patients as partners in their care. Our results did not mention anything related to the ‘change of culture’ in the definition. According to the literature, the inclusion of cultural change in the concept is important to the extent that disregarding it can make research conceptually and theoretically limited [39]. The literature also emphasizes that not all patients want to participate in activities other than those related to their care, and ‘willingness’ and ‘availability’ were requirements for participation at any level of engagement. From the perspective of institutions, on the other hand, some of them have barriers and restrictions on the sharing of information, especially in critical situations involving ‘care risk, legal risk or even image risk’ [15].

Regarding how engagement is performed, the terms direct to communication as the basis of the engagement process. The model developed by Rowe and Frewer [40] defines the types of engagement from the flow of information that range from a unilateral flow to the dialogue between patients and healthcare professionals. These terms also refer to the patient’s perspective, based on his or her experience [2, 23, 41] and on his or her opinion and willingness as a patient-consumer [5], reinforcing the concepts from the literature.

According to Catlow et al. (2021), negative feedback without a plan to improve, risks reducing confidence and impeding performance, as does increased anxiety. Conversely, improved patient experience may reduce harm [42]. Patient engagement had beneficial effects and unintended or harmful impacts, such as overextended patient/family advisors, patient/family advisor turnover and clinician frustration if PFE slowed the pace of planning and improvement [13].

In terms of where or on what engagement takes place, two levels of involvement were observed in the concept—individual care and organizational improvement level, similar to that presented in the literature [4, 29]. This reinforces the statement that the concepts 'centered care' and 'participation' are not independent and should be seen as interrelated [4] and the evolution of the paternalistic process to an approach in which the patient's experience must be recognized [43]. Information about patient’s experience and their feedback can be used as an educational tool and as an integral component of quality improvement and professional development to improve medical performance [44].

The final part of the definition refers to the results or "why engage". In the construction of the definition, two objectives were observed: directing actions to what truly matters [45] to the P/F and ensuring the quality of services and patient safety. The contribution from the patient's perspective in quality improvement initiatives strengthens the change required to achieve the third era of quality evolution in the health area, proposed by Berwick [1]. The interrelation between quality and safety attributes also reinforces the literature [46]. Physicians can use communication behaviors as they seek to improve patient participation and decrease malpractice risks [47].

Comparing the answers focused on the individual and organizational levels, it was observed that they were different in the dimensions of what, where, why and how. Most of the respondents described engagement at the individual level. This should explain why Brazilian hospitals remain in an early stage of patient involvement in quality programs [35]. Meaningful engagement requires reflection on the reasons and objectives for patient involvement and the preparation and support needed for successful changes [48].

There are some studies defining and/ or discussing engagement in depth based on theoretical foundations which for this topic can be highlight consumerism [49, 50], humanist considerations [5], standpoint theory [41], strong objectivity [41], feminist theory [41], power of symbolic capital [39, 51], concept of participatory parity [39, 51] and emancipatory, democratic or technocratic models [39, 51]. As mentioned in the Introduction, the motivation for carrying out this study was the inconsistency and lack of consensus about the concept of 'patient engagement', although much has been studied on the subject, its mechanisms, and intervening factors at different levels of care. This research contributes since it presents the health professionals’ point of view who are in hospitals and are responsible for the decision to engage and, if so, how to do it. Understanding their points of view and how this interferes in practice may be important to get the knowledge gap.

It is important to note that patients can also be involved in healthcare research, and patients can be partners throughout the research process or in some specific tasks [45]. Based on a systematic literature review, Harrington et al*.* (2020) defined patient engagement specifically in research [52].

## Final considerations

Involvement, participation, and centered care were considered synonymous with engagement. The participants described patient participation at both the individual (related to the treatment and decision-making about the therapeutic plan) and organizational levels (related to quality improvement). The PFE in the treatment is related to the development, discussions and decision-making about the therapeutic plan, participation in each step of care, and knowledge of the institution's quality and safety processes. At the organizational level, engagement in quality improvement includes the involvement of the P/F in all processes of the institution, from strategic planning to the design or improvement processes, as well as active participation in institutional committees or commissions.

The results suggest that there is a relationship between the respondents' point of view about what engagement means and the level of engagement performed by institutions according to the mechanisms they have implemented. Hospitals that implement mechanisms to consult the opinion and voices of patients defined engagement at the individual level. On the other hand, hospitals that implement mechanisms that allow dialogue between professionals and patients, characteristic of the level of involvement, defined PFE at the organizational level.

This research presents some limitations, highlighting a non probabilistic and convenience sampling plan limiting the generalizability of results to the population, and the conclusions refer only to the elements participating in the research. However, it is important to emphasize that this was an exploratory study. The low rate of response may also have introduced a bias in the results. The lack of response from many hospitals might have led to self-selection. Perhaps this topic is more advanced in theory within academia than in practice among professionals in institutions. Finally, the findings may not be relevant to the hospitals in the sample that do not perform educational function, because there were few respondents.

A suggestion for future studies would be to confirm the hypothesis whether there is a relation between the concept of engagement from the point of view of the institution's professionals and the level of patient engagement.

## Supplementary Information

**Additional file 1: Appendix 1.**

## Data Availability

The datasets used and/or analyzed during the current study are available from the corresponding author upon reasonable request.
