# Supplementary material for: How did healthcare professionals define patient engagement in quality management? A survey study
Source: BMC Health Serv Res. 2023 Feb 20;23:173. doi: 10.1186/s12913-023-09098-z (PMC9942306; doi:10.1186/s12913-023-09098-z)
Supplement: Supplementary file 1 — Additional file 1: Appendix 1. [file 12913_2023_9098_MOESM1_ESM.docx]

**Appendix 1**

1. How would you define 'patient and family engagement in quality management'?
2. At your institution, what synonyms are used for patient engagement?

| ( ) Activation | ( ) Collaboration | ( ) Coproduction |
| --- | --- | --- |
| ( ) Centered care | ( ) Empowerment | ( ) Involvement |
| ( ) Partnership | ( ) Participation | ( ) None of the options |

1. Considering that 'engagement mechanisms' are processes, methods, techniques and tools used to allow the participation of patients and family members. Please identify below the mechanisms for engaging patients and family members in quality management that are used in your institution.

| Mechanisms of engagement | Not implemented | In progress | Implemented in one or a few units | Implemented |
| --- | --- | --- | --- | --- |
| P/F satisfaction survey (continuously) |  |  |  |  |
| P/F satisfaction survey (annual, by sampling) |  |  |  |  |
| P/F experience survey (continuously) |  |  |  |  |
| P/F experience survey (annual, by sampling) |  |  |  |  |
| Ad hoc research (punctual and with specific objectives) |  |  |  |  |
| Formal communication process in relation to doubts, suggestions, complaints, and compliments |  |  |  |  |
| Suggestion box |  |  |  |  |
| Interview for root cause analysis |  |  |  |  |
| Panel or focus groups |  |  |  |  |
| Member of the improvement project team |  |  |  |  |
| Member of the research project team |  |  |  |  |
| Member of the root cause analysis team |  |  |  |  |
| Member of the Quality or Management Committee |  |  |  |  |
| Member of an Advisory Board |  |  |  |  |
| Member of the Board of Directors |  |  |  |  |
| Shares the leadership of Safety and Quality Improvement Committees |  |  |  |  |
| Development of process standards, tasks, or protocols |  |  |  |  |
| Preparation of booklets or other materials for communication with patients |  |  |  |  |
| Evaluation of quality goals and/or objectives |  |  |  |  |
| Development of quality criteria |  |  |  |  |
| Content development for training others P/F |  |  |  |  |
| Educator in the training of others P/F |  |  |  |  |
| Content development for training professionals |  |  |  |  |
| Educator in the training of professionals |  |  |  |  |
